# The “common” experience of voice-hearing and its relationship with shame and guilt: a systematic review

**DOI:** 10.1186/s12888-022-03902-6

**Published:** 2022-04-20

**Authors:** E. Volpato, C. Cavalera, G. Castelnuovo, E. Molinari, F. Pagnini

**Affiliations:** 1 Present Address: Department of Psychology, Università Cattolica del Sacro Cuore, Largo A. Gemelli, 1, 20123 Milan, Italy; 2 IRCCS Fondazione Don Carlo Gnocchi, Milan, Italy; 3 Istituto Auxologico Italiano IRCCS, Psychology Research Laboratory , Verbania , Italy; 4 Laboratory, Istituto Auxologico Italiano IRCCS, Milan, Italy; 5 Department of Psychology, Harvard University, Cambridge, MA USA

**Keywords:** Auditory hallucinations, Shame, Guilt, Continuum approach, Mental health

## Abstract

**Background:**

Despite Auditory Verbal Hallucinations (AVHs) having been long associated with mental illness, they represent a common experience also in the non-clinical population, yet do not exhibit distress or need for care. Shame and guilt are emotions related to one's perception of oneself and one's responsibility. As such, they direct our attention to aspects of AVHs that are under-researched and elusive, particularly about the status of voices as others, their social implications and the constitution and conceptualisation of the self.

**Objectives:**

This paper aims to provide a systematic review of studies that investigated the relationship between auditory hallucinations, shame, and guilt in people without relevant signs of psychiatric issues.

**Methods:**

We searched studies reporting information about voices characteristics, the relationship between voices and hearers, hearer's reactions, and beliefs, paying peculiar attention to shame and guilt issues. Included papers were evaluated for risk of bias.

**Results:**

Eleven studies that explored the relationship between AVHs, shame and guilt, were extracted. Phenomenological, pragmatic, as well as neuropsychological features of hearing voices in non-clinical populations, allowed us to note a dynamic relationship and the constellation of subjective experiences that can occur. The role of guilt was characterized by few studies and mixed results, while shame was mainly common.

**Conclusions:**

Due to the high heterogeneity detected and the scarce sources available, further studies should focus on both the aetiology and the bidirectional relationship between hearing voices, shame, and guilt in non-clinical people. This can be helpful in therapies for non-clinical populations who are distressed by their voices (e.g., psychotherapy), and for whom shame, and guilt may contribute to negative consequences such as isolation, anxiety or future depression. Moreover, it might favour the development and implication of different treatments considering emotion regulation, distress tolerance and interpersonal sensitivity on the clinical populations.

**Supplementary Information:**

The online version contains supplementary material available at 10.1186/s12888-022-03902-6.

## Background

Auditory Verbal Hallucinations (AVHs) are often associated, in psychiatric contexts and everyday life, with the universe of mental illness. Although there is not a univocal consensus concerning the definition, the DSM-5 delineates hallucinations as perceptions-like experiences that others would not sense and do not exist outside one's perception (DSM-5).

According to Romme and colleagues [1] hallucinations are a disorder of perception that people describe as being located in the external world (ego-dystonic), with the same qualities of “normal” perceptions, but in the absence of external stimuli [2]. Reported hallucinations are perceived as real, as demonstrated by Aggernaes’s study [3], later confirmed by Barrett and Caylor [4] and Garrett [5].

AVHs indicate auditory perceptions of speech [6], with pragmatic properties similar to “real” speech [7]. In healthy people, AVHs are isolated experiences that can be associated with psychological conditions such as anxiety, depression, difficulty concentrating, suspiciousness or withdrawal from family and friends [8]. They can experience voices principally because of sleep deprivation or major life stressors and can be intra- or extracranial [9]. Voices are often related to individuals’ lives, and they are often described as messengers [10], communicating something that can be very important for hearers (i.e. may be acting as a voice of their conscience). Despite some voices may be hushed, loud or difficult to discern, the main part of the hearing sounds is audible [11, 12]. AVHs mainly comprise someone’s else voice or multiple sounds [11], familiar [13] as well as unfamiliar voices [12].

The extent and presentation of AVH may be different within individuals in need of care, and there have been proposals for undertyping AVHs. Conceptually, there has also been a distinct shift away from categorical models towards a view of the continuum of psychotic symptoms and abnormal experiences that extends not only across diagnostic categories but also into the non-clinical population [14]. Conceptualisations of the continuum mostly refer to psychosis or schizotypal personality traits in the general population, so their relevance to AVH in healthy individuals is all but inferred. According to the most widespread epidemiological conceptualisation of extended and transdiagnostic phenotypes with latent subgroups, both quasi- and full-dimensional models would fit. According to the diagnostic model, in fact, non-harmful AVHs would differ in experience from those found in the clinical population with AVHs, and persons considered "healthy" who hear voices and "healthy" populations who do not should be indistinguishable on almost all parameters (e.g., exposure to risk factors). According to the quasi-dimensional model, non-clinical populations that hear voices will be somewhere in between the clinical populations that hear voices and the healthy population on almost all parameters, including need for care and voice-distress. In such a model, increased psychotic experiences would be associated with expanded need for care. Finally, a fully dimensional model would predict that the occurrence of AVH is largely unconnected to need for care, and non-clinical voice hearers should be at no greater risk of distress than the general population. The other parameters should vary arbitrarily. Non-clinical individuals are more likely to appraise their hearing voice’s experience as benign, positive, and controllable [15], less hallucination-related distress [16], and with lower levels of negative beliefs about AVHs than people with psychiatric disorders [9, 17, 18]. Therefore, hearing voices cannot be considered always as uniformly pathological or with being related to situations characterized by high need of care. Even in the case of voices related to negative emotional experiences, the content of the message is usually characterized by a contained level of hostility and negativity not to elicit severe states of impairment [9]. Healthy individuals develop different beliefs about the origins of the voices (from within myself in 41% vs. from others in 42% cases), 17% are uncertain if these voices are real or not [19] and others believe that they originate from guardian angels, source of deceased and ghosts. Indeed, while non-clinical voice-hearers more frequently endorsed external or non-specific spiritual explanations about the origins of the voices heard, clinical voice-hearers more frequently explained that the voices were other (living) people, God, demons/evils or implanted devices [14, 19].

Some epidemiological studies tried to provide an estimate of the frequency of AVHs in the general population but they neither give any indication of the quality of these experiences nor do they clarify whether they are comparable to the AVHs experienced by those diagnosed with a clinical disorder [14, 20]. Studies that have investigated the distribution of hearing voices in the general population have used different instruments and reported results differently. For this reason, there is limited information on how common the experience of non-clinical AVHs is in different age groups, such as in males and females. The prevalence of voice-hearing in non-clinical adult populations is about the same as in children, ranging from 10 to 15%. Sidgewick, Johnson, and Myers (1894) conducted the first reported survey on verbal hallucinations, collecting data from approximately 17,000 participants, in 10 countries; they found that approximately 7.8% of men and 12% of women reported hallucinations (3.3% auditory hallucinations). Linscott & van Os, considering studies on children and adolescents, found a prevalence of 7.2% [21]; while Kelleher et al. a higher median prevalence of 17% in children (9–12 years) compared with 7.5% in adolescents (13–18 years), suggesting how, while psychotic symptoms may be more commonly experienced during a child's typical development, they become less frequent and increasingly indicative of pathology as the child grows older [22, 23]. Recent meta-analyses estimated both a lower median prevalence of psychotic experiences of 6% and a median incidence of 1.2% in the general population [21], indicating that age, minority or migrant status, marital status, income, education, alcohol and cannabis use, stress, urbanization and family history could all influence their onset [17]. Furthermore, it can be difficult to identify and recruit nonclinical voice-hearers, because it may be uneasy to share their own experience, probably fearing encountering stigma [18, 24] or to be isolated by other people [25–27]. Consequently, it is not easy to correctly estimate the prevalence of the phenomenon.

### Shame, guilt and AVHs

Shame and guilt are self-conscious emotions that convey crucial information in the way we consider ourselves and they can both regulate social behaviours in interpersonal situations. Literature of the last two decades agrees on the fact that shame and guilt are two separate emotions with different implications for psychological adjustment [28, 29]. While the feeling of shame is linked to a stable internal and negative perception of the entire self (e.g., “I’m a bad person), guilt appears when people attribute an adverse outcome to unstable causes (e.g., “I’ve made a bad mistake”). Therefore, shame is associated with feelings of inferiority and worthlessness for a defective self while guilt is associated with feelings of tension and remorse [30]. They also involve different behavioural trajectories: while shame is associated with the desire to escape the disappearing of others, guilt is related to the desire to apologize, confess, or repair. While the social function of guilt is linked with reparative behaviours, the adaptive role of shame is to communicate/show similar patterns of brain activation related to the temporal lobe, but while guilt seems related to the amygdala and insula, shame is involved in the frontal lobe activation [31].

### The relationship between shame and guilt and mental disorders

The relationship between shame and guilt and mental disorders has been explored in the last decades. Both shameful feelings of powerlessness and maladaptive feelings of guilt and hyper-responsibility can exacerbate anxiety, depression, eating behaviours and traumatic symptoms [32]. Even if the different studies showed that shame can be more strongly associated with various psychological symptoms [33–35], still little is known about how shame and guilt are involved in the perception of AVHs. As evidenced by McCarthy-Jones (2017), the occurrence of AVHs may be linked with the use of inflexible approaches aimed at processing unresolved events related to shame- and guilt-related experiences. Inflexible avoidant strategies such as dissociation and suppression that are commonly used to deal with shame- and guilt-related events often cause a rebound effect that can generate intrusive AVHs with unexpected greater vigour [36]. Within this perspective AVHs related to shame and guilt can be considered in a continuum of clinical severity: the more inflexibility is adopted to try to deal with negative experiences the more AVHs will be severe as they will be probably related to unresolved materials and ruminative thoughts [37]. On the contrary, the use of a flexible “talking to myself” strategy can foster adaptive meanings of past negative experiences. This can avoid cognitive or emotional overload and enhance a constructive internal dialogue.

### Objectives

Despite a certain level of knowledge about auditory hallucinations in psychiatric populations, in the last decades, there have been few reviews about AVHs in a non-clinical sample, and they were limited in scope (e.g., they focused on specific mechanisms or experience) [38], theoretical, centred on the continuum [14] or focused on the comparison between clinical and non-clinical voice-hearers [20, 39–42].

Moreover, in the previous studies, auditory verbal hallucinations were mainly described as related to cognitive cause and consequences, while the potential involvement of emotions such as shame and guilt was neglected. Shame and guilt are unpleasant emotions that allow to regulate the social interactions and are not necessarily characterized by a clinical need of therapeutic care. They can both help us to understand our mistakes and to identify when expectations of our idea of ourselves are not been met. In some cases, shame and guilt can both contribute to unpleasant consequences on long-term mental health outcomes and on listening to voices themselves and contribute to adverse consequences such as isolation or increased rates of depression, anxiety or other mental disorders. A more systematic exploration of how shame and guilt may influence or be influenced by listening to voices in non-clinical people is therefore needed. Despite the crucial information in the way we consider ourselves, shame, guilt, and the intrusive thoughts that are often related to these emotions, no review explored their relationship with AVHs.

This systematic review aims to describe and tease out the relationships between non-clinical voice-hearers, AVHs, shame and guilt. Specific objectives include understanding whether guilt and shame are related to the negative or positive aspects of listening to the voice, to be able to clarify and, possibly, outline differentiated interventions for those non-clinical listeners who experience the consequences of these emotions in their daily lives.

## Methods

### Registration and protocol

The guidelines of the Preferred Reporting Items for Systematic reviews and Meta-Analyses (PRISMA) were followed [43]. The study protocol was registered with PROSPERO and the registration number is CRD42017054819.

### Eligibility criteria

Papers were included if they were published in English in a peer-review journal up to July 27^th^, 2021, and met the criteria outlined in Table 1. We decided to also include studies concerning interventions that considered non-clinical populations, or that concerned a comparison between non-clinical and clinical populations, if any, to be in line with the proposed objectives.

**Table 1:** Inclusion and exclusion criteria

Criterion	Inclusion	Exclusion
Time period	1946-2021	Studies outside these dates
Language	English (recognized language of international scientific debate)	Non-English
Type of article	Original research, publishedin a peer review journal. Qualitative or quantitative studies (Randomized Controlled Trials (RCTs), uncontrolled open trials that involved a comparison between at least two groups or a pre-post study design and cross-sectional studies); commentaries, letter, editorial	Articles that were not peer reviewed, only abstract avalaible
Ethics clearance	Studies with approved ethics notification	Studies without approved ethics notification
Study focus	Todescribe and tease out the relationships between non-clinical voice hearers, AVHs, shame and guilt.	Studies that don't consider the relationship between AVHs, shame and guilt in non clinical samples
Population and sample	Adults, adolescents or childs that were not in contact with mental healthcare services because of hearing voices or they were at a first visit for this problem. We also considered studies in which healthy voice-hearers constituted a control group in comparison with psychiatric patients.	All the other chronic diseases or psychiatric conditions
Types of study design	We included all Randomized Controlled Trials (RCTs), uncontrolled open trials that involved a comparison between at least two groups or a pre-post study design and cross-sectional studies. Since we would like to have examined all available literature, we chose to include both controlled and uncontrolled studies, rather than restricting our analysis to RCTs. Case studies, case series studies and qualitative studies were also included.	All the other kinds of study design
Types of interventions	Face-to-face clinician-delivered treatment, computer-delivered treatments, and cognitive tasks. These latter were included both if they were guided by a clinician (i.e., he/she supported person during the intervention or he/she read the instructions to the task) and when the participants were invited to fill out a questionnaire on a Web Site.	All the other kinds of interventions
Types of comparisons and outcomes	Self-report, clinician or proxy administered psychometric instruments that evaluated Auditory Verbal Hallucinations (AVHs) or an interview about this issue and at least one measure about shame and/or guilt. Diagnostic status was also considered to exclude all studies about only psychiatric patients. We also consider outcomes reported qualitatively.	Diagnostic status was also considered to exclude all studies about only psychiatric patients.

### Search strategy

To identify studies for possible inclusion, we conducted comprehensive searches of the electronic databases PsycINFO and PsycARTICLES, MEDLINE, PubMed, EMBASE Classic (1947-1973) and EMBASE (1974-2021), Web of Knowledge, Scopus and The EBSCO Online Research Database, Epub Ahead of Print, In-Process & Other Non-Indexed Citations, Ovid MEDLINE(R) Daily and Ovid MEDLINE(R) (1946 to Present), Global Health (1973 to 2021), HMIC Health Management Information Consortium (1979 to July 2021), Journals@Ovid Full Text, Mental Measurements Yearbook (1st to 20th Yearbooks March 2021), PsycARTICLES Full Text, PsycINFO (1806 to July 2021), Social Policy and Practice, Your Journals@Ovid up to 27^th^ July 2021 (Table 2).

**Table 2:** Key words adopted for the search strategy

**Key words**	
*“*Auditory Verbal Hallucinations”, OR “AVH”, OR “Auditory Hallucinations”, OR “Verbal Auditory Hallucinations”, OR “VAH”, OR “hearing voices”, OR “voice-hearing” AND “non-clinical population”, OR “non-clinical patients”, OR “non-patients”, OR “non-psychosis”, OR “non-psychiatric”, “general population”, OR “normal people” AND "shame" OR "guilt".	

The findings were cross-referenced with references from reviews.

### Study screening and selection

Following electronic searches, retrieved records were uploaded to Mendeley where duplicates were removed. After a preliminary training exercise inconsistently using inclusion and exclusion criteria, two reviewers (EV; CC) independently selected potentially eligible papers. The final set of records was selected by reading the full texts of all articles that passed an initial screening of titles and abstracts. Disagreements were resolved thanks to a consensus with a third reviewer (FP).

**Fig 1:**
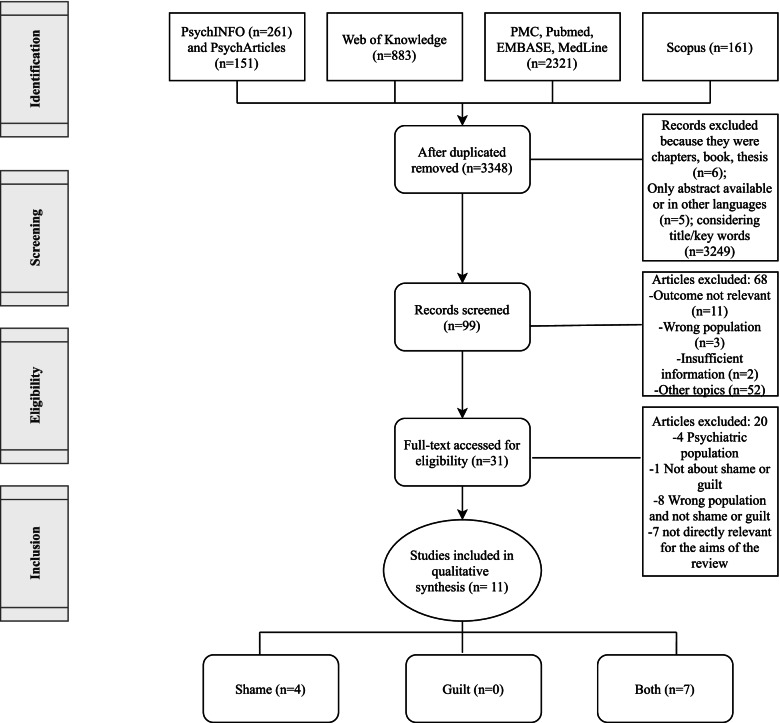
PRISMA Flow chart diagram of the selection process

Figure 1 shows a flow chart diagram of the search process. Through electronic and manual searches, 3348 separate records were identified after duplicates were removed. Ninety-nine records were screened, and 31 relevant papers were downloaded for full-text screening. A final set of 11 papers, which met inclusion and exclusion criteria, were selected for review.

### Data extraction, study quality assessment and synthesis

A structured template was developed to extract relevant data from eligible papers. Four reviewers (EV; CC; FP; GC), independently, extracted data and conducted study quality assessment, using a codebook. Discrepancies between researchers were resolved through discussion.

Data were extracted on publication date, type, country, participant characteristics (such as age, gender, recruitment method, kind of psychiatric illness), AVH (according to their pragmatic and phenomenological features), details of assessment instruments, number of cases, setting, dropout rate, physiological parameters, the presence of a family member, explored themes, follow-up.

The methodological quality of each study included in the systematic review was assessed using a standard form adapted from the Mixed Methods Appraisal Tool (MMAT) –Version 2018, a critical appraisal tool that is designed for the appraisal stage of systematic mixed studies reviews [44]. The four criteria were selection bias, quality of measurement, adjustment for confounders and proportion of complete outcome data/response rate/follow-up rate (see Supplementary file). We conducted a narrative synthesis of the results because the expected heterogeneity of the included studies, e.g., in samples, predictive measures and outcomes, made a meta-analysis inappropriate.

## Results

### Study characteristics

Table 3 shows the main characteristics of the included studies. We identified a total of 11 studies. Among the identified trials, there are 3 (27.3%) cross-sectional studies or surveys, 1 (9.09%) cohort study, 3 (27.3%) reviews, 2 (18.2%) hypothesis and theory articles, 1 (9.09%) qualitative research and 1 (9.09%) original article. The studies were published between 2017 to 2019. They were published in UK (3, 27.3%), USA (2, 18.2%), Australia (2, 18.2%), Ireland (1, 9.09%), Norway (1, 9.09%), France (1, 9.09%), The Netherlands (1, 9.09%).

**Table 3:** Characteristics of the studies included

Title	Authors	Journal	Year	Country	Kind of Study	Tot. Subjects	Gender (M/F)	Mean Age	Mean Time from diagnosis (months)	Kind of psichiatric illness	Objectives
Compassion Focused Approaches to Working With Distressing Voices	Heriot-Maitland C, McCarthy-Jones S, Longden E, Gilbert P.	Frontiers in Psychology	2019	UK	An outline of voice-hearing phenomenology in the context of evolutionary mechanisms for self- and social- monitoring.	N/A	N/A	N/A	N/A	N/A	To provide an outline of voice hearing phenomenology within the compassioned focused approach
Shame, social deprivation, and the quality of the voice-hearing relationship.	Carden LJ, Saini P, Seddon C, Evans E, Taylor PJ.	Psychology and Psychotherapy: Theory, Research and Practice	2019	UK	Online survey	171	50/21	37.8 (12.4)	Not reported	General population	to explore the possible psychosocial determinants of the relationship between the hearer and the voice, focusing on shame and social deprivation as putative correlates of voice relationship.
The ice in voices: Understanding negative content in auditory-verbal hallucinations.	Larøi F, Thomas N, Aleman A, Fernyhough C, Wilkinson S, Deamer F, McCarthy-Jones S.	Clinical Psychology Review	2019	Norway	Review	N/A	N/A	N/A	N/A	N/A	
On shame and voice-hearing.	Woods A.	BMJ Med Human	2017	UK	Original article, case studies	2	1/1	range 20-40	Not reported	PTSD, schizoaffective disorder	To focus on shame and voice-hearing, taking as its point of departure the testimony of two voice-hearers
Affective and cognitive factors associated with hallucination proneness in the general population: the role of shame and trauma-related intrusions.	Bortolon C, Raffard S.	Journal of Cognitive Neuropsychiatry	2018	France	Survey	179	33/146	24 (6.61)	Not reported	Childhood trauma	To explore the mediation role of shame, trauma-related intrusions and avoidance in the association between childhood trauma and hallucination-proneness.
Is Shame Hallucinogenic?	McCarthy-Jones S.	Frontiers in Psychology	2017	Ireland	Hypotheis and Theory paper	N/A	N/A	N/A	N/A	N/A	N/A
Stigma and need for care in individuals who hear voices	Vilhauer, R.P.	International Journal of Social Psychiatry	2017	USA	Review	N/A	N/A	N/A	N/A	N/A	N/A
Hallucinations in Healthy Older Adults: An Overview of the Literature and Perspectives for Future Research	Johanna C. Badcoc	Frontiers in Psychology	2017	Australia	Review	N/A	N/A	N/A	N/A	N/A	N/A
Auditory hallucinations, top-down processing and language perception: a general population study	de Boer, J.N.	Psychological Medicine	2019	The Netherlands	Online survey	5115	no HV: 60.5% female; AH ever: 68.6%; AH month: 74.4%; AH week: 74.3	No HV: 38.8; HV ever: 36.1; HV month: 33.2; AH week: 33.44	Not reported	General population	To explore the relationship between the occurrence of auditory hallucinations (AH) and the strength of top-down processes in auditory language perception
Perspectives on the Delirium Experience and Its Burden: Common Themes Among Older Patients, Their Family Caregivers, and Nurses	Eva M. Schmitt et al.	The Gerontologist	2017	USA	Qualitative interview study	18 patients who had recently experienced a delirium episode	77/11	Patients (n = 18) had a mean age of 79 years (range 70–92)	Not reported	8 (45%) reported a previous delirium episode that was con-firmed in the medical record, and 2 (11%) self-reported their previous episode	To describe common delirium burdens from the perspectives of patients, family caregivers, and nurses.
A comparison of hallucinatory experiences and their appraisals in those with and without mental illness	Connell M., Scott J., McGrath J., Waters F., Laroi F., Alati R., Najman J., Betts K.	Psychiatry Research	2019	Australia	Cohort Study	253 (43 no mental disorder VS 210 mental disorder (psychiatriac or not)	31%/69%	30-33 years	Not reported	Mental disoders VS no Mental D	To compare the characteristics of voice-hearing, and their appraisals, in individuals with psychotic disorder, non-psychotic mental disorder and no disorder in the general population.

Title	Outcomes	Setting	Follow up	Drop-out rate	Inclusion Criteria	Exclusion Criteria	Analysis	Instruments	Physiological parameters	Family member	Explored Themes	Findings	QL/QNT
Compassion Focused Approaches to Working With Distressing Voices	N/A	N/A	N/A	N/A	N/A	N/A	N/A	N/A	N/A	N/A	N/A	Compassion focused therapy allows to identify the threat-based (dominant-subordinate) motivational systems when they arise. Moreover, it could play a role in understanding their function in the context of life, and shift into different motivational patternsthat are orientated around safeness and compassion.	N/A
Shame, social deprivation, and the quality of the voice-hearing relationship.	Hearing voice	Online	None	Nine participants left prior to completing the study, four of whom did not complete any of the questionnaires and five participants that did not continue past the first questionnaire	Participants must have heard at least one voice, irrespective of any mental health diagnosis. Individuals hearing a single voice or multiple different voices were both eligible for the study. The voice(s) must have occurred for at least 1 month and must have been a current experience at the time of participation. The voice(s) could produce a word or words, but also other utterances that could be attributed to a being (e.g., laughing, crying). Other auditory hallucinations that could not be related to an individual (e.g., machine noises) were not classed as a voice. The voice(s) may have been perceived as human or non-human (e.g., god) or viewed as a product of psychosis or illness. Only participants aged 18 and above, who lived in England, and who could understand or speak English were eligible for recruitment into the study.	Not specified	Nonparametric Spearman’s correlational analyses; a principal component analysis (PCA); a multiple linear regression	The Experience of Shame Scale (ESS); IMD score; Beliefs About Voices Questionnaire-Revised (BAVQ-R); Voice and You scale (VAY); Positively framed relational items to accompany the Voice and You scale	None	None	Relationships between social deprivation, shame, and the voice- hearing variables	Social deprivation and shame were not associated. Shame was positively associated with variables describing negative voice-hearing beliefs/relationships but not positive voice-hearing beliefs/relationships.	QNT
The ice in voices: Understanding negative content in auditory-verbal hallucinations.	N/A	N/A	N/A	N/A	N/A	N/A	N/A	N/A	N/A	N/A	N/A	Different variables and factors may drive the nagative content of AVHs. In particular, adverse life-events may underpin much negative voice-content. This relationship could be mediated by mechanisms such as hypervigilance, reduced social rank, shame and self-blame, dissociation, and altered emotional processing	N/A
On shame and voice-hearing.	N/A	N/A	N/A	N/A	N/A	N/A	N/A	Non-structured interview	N/A	None	Phenomenological and psychological features of shame in voice-hearing	Thanks to the testimonials, this articles stresses the relevance to pay attenttion to both the psychological and phenomenological features of voice-hearing in relation to shame, social cognition and the conceptualisation of the self.	N/A
Affective and cognitive factors associated with hallucination proneness in the general population: the role of shame and trauma-related intrusions.	N/A	Online	None	None	being able to speak and understand French and being older than 18 years old and younger than 60	4 persons excluded because older than 60s	Mean, standard deviation and frequency of socio-demographic and clinical variables were calculated. Multiple regression analyses	Adverse childhood experiences questionnaire (ACE); Impact of event scale-Revised (IES-R); Experience of shame scale (ESS); The Launay–slade hallucination scale (LSHS)	None	None	[1] trauma (independent variable- IV) was associated with hallucination-proneness (depen- dent variable- DV) [2]; the IV was associated with shame, intrusions, and avoidance (mediation variables – MV) [3]; both the IV and MD were associated with the DV. If stat- istically signi!cant associations were found for step [2] and [3], we could conclude that there is an indirect e"ect.	Having intrusive thoughts and memories about past negative experiences together with feelings of shame may contribute to hallucination-proneness following childhood trauma	QNT
Is Shame Hallucinogenic?	N/A	N/A	N/A	N/A	N/A	N/A	N/A	N/A	N/A	N/A	N/A	One of the main hypothesis is that, reducing shame, the shamful content of AVH will be reduced. Moreover, shame will mediate the relationship between trauma and AVH and it will influence the treatments' efficacy. Another hypothesis stresses the idea that shame plays a key role in the onset of AVH after a trauma	N/A
Stigma and need for care in individuals who hear voices	N/A	N/A	N/A	N/A	N/A	N/A	N/A	N/A	N/A	N/A	N/A	Stigma contribute to making voice hearing experiences pathognomonic	N/A
Hallucinations in Healthy Older Adults: An Overview of the Literature and Perspectives for Future Research	N/A	N/A	N/A	N/A	N/A	N/A	N/A	N/A	N/A	N/A	N/A	The emergence of hallucinations is as a balance between the sensory, cognitive, or social impairments accompanying advancing age and the degree to which compensatory processes elicited by these impairments are successful	N/A
Auditory hallucinations, top-down processing and language perception: a general population study	Number of responses on distracting cues	Online	None	2079	[1] being a native speaker of Dutch (to avoid differences in perception based on language fluency), and [2] age of 14 and over.	Not specified	Participant characteristics were compared between groups using a χ2 test for categorical values and an analysis of variance for continuous variables. A Kruskal–Wallis test was used in case an assumption was violated. Relevant test assumptions were assessed visually by evaluating Q–Q plots of the residuals and scatter plots of the predicted values and the unstandardized residuals. A general linear model (GLM) was also applied.	Online auditory verbal recognition task; Stimuli selection and recording; Questionnaire for Psychotic Experiences	Hearing test	None	N/A	Individuals with AVH are less sensitive (i.e. reduced discrimin- atory ability) in their auditory word recognition, as expressed by a higher false alarm rate compared with individuals without AH	QNT
Perspectives on the Delirium Experience and Its Burden: Common Themes Among Older Patients, Their Family Caregivers, and Nurses	N/A	Clinic	1 month after discharge	None	Inclusion criteria for patients included age 70 years or older, admission to the general medicine service of a large, urban, acute-care academic medical center, ability to communicate effectively in English (including adequate hearing), residence within a 40-mile radius of the hospital, and a positive screen for delirium.	patients with dementia; active alcohol abuse, diagnosis of schizophrenia or active psychosis, developmental delay, and terminal condition.	They digitally recorded and professionally transcribed all interviews	Semistructured qualitative interviews about delirium burden	None	16 family caregivers, and 15 nurses who routinely cared for patients with delirium	The common themes across patients, nurses and caregivers were: Symptom Burden, Emotional Burden, and Situational Burden.	Delirium is a shared experience between patients, caregivers and nurses and it is linked to the burden's experience	QL
A comparison of hallucinatory experiences and their appraisals in those with and without mental illness	N/A	Clinic	N/A	Not reported	Not specified	Not specified	Correlations; regression analysis	Structured Clinical Interview for DSM-IV Axis I Disorders (SCID-1); Appraisals of Anomalous Experiences Interview (AANEX)	None	None	N/A	Those with a non-psychotic disorder had more than twice the odds of voice-hearing compared to those with no disorder. Vioce-hearing and their negative appraisals were similar between groups and their recurrence differentiated clinical from non-clinical populations.	QNT

#### The relationship between shame and AVH

Shame was found to be positively correlated with more variables describing negative features of verbal hallucinations in non-clinical populations [45]. Specifically, adverse life events may prompt greater onset of negative content, characterised by a relationship mediated by mechanisms including those involving shame and guilt [36, 46, 47]. According to some studies, AVH may play a defensive role by facilitating shame experiences in traumatised people to encourage self-protective behaviour to aid survival. In such an account, AVH could be considered an evolved mechanism to protect traumatised people from further harm [36]. At the same time, intrusive memories, images and thoughts about past experiences, together with the shame associated with them, can stimulate the onset of traumatic experiences [36, 48]. Furthermore, a couple of studies underline the need to find functional interventions that reduce stigma rates towards those who hear voices [24], taking into account differences linked to the life cycle [49]. Moreover, de Boer's study claims that, once in a lifetime, the experience of an auditory hallucination is associated with an aberrant perception of auditory language, suggesting a close relationship between the two processes in the general population [50].

#### The relationship between guilt and AVH

Although their relationship with pathological disorders, guilty AVH in non-clinical population can also have the role of establishing a precise responsibility related to personal negative experiences. Therefore, AVH that show guilty contents may also give a perception of order towards stressful events that otherwise would seem even more frightening and chaotic. This can give the transgressor a sense of control over negative events and help avoid similar mistakes in the future. AVH of guilt can be seen as an internal reminder of past mistakes that can be viewed as the source of helpful albeit challenging messages. Within this perspective, AVH of guilt can be related to an internalized voice of moral authority that can guide interpersonal behaviour enhancing social relationships and restoring equities. Furthermore, in contrast to shame, guilt is perceived by the hearer as a burden on those around him, especially in the case of older adults. People feel guilty about experiencing the voices, fearing that they will become a cause of concern for their loved ones or that they will be mocked or not understood [51]. In this sense, the relationships between guilt and shame are unclear. These results are limited to the few studies found and included in this systematic review, denoting the need for further investigation.

### Risk of bias in studies

The quality of included studies is generally low and most of them received just a first screening approval because their overall quality scores were not in complete accordance with the methodological quality criteria of MMAT (Supplementary Material 1). Indeed, many studies did not have complete information about baseline response rate, outcome data, follow up and none of them was a randomized controlled trial, resulting in a lower quality assessment rating. This makes it impossible to conclude the relationship between shame, guilt, and auditory hallucinations in the non-clinical population, although it can be assumed that there is a circular relationship, probably also influenced by the phenomenological characteristics of the voices themselves.

## Discussion

### Shame and AVH in a non-clinical population

The experience of hearing voices can be correlated with shameful experiences in several ways. Like other emotions such as sadness, shame may have a role in shaping voices and activating them in specific circumstances [36]. Firstly, AVH can represent a source of significant distress in terms of content, meaning ascribed, and the relationship between the voice and the hearer. Shame was found to be related to the negative content, qualities of the heard voices and the subjective ratings of deprivation [45]. This finding supports the idea of mirroring between voice hearers’ experience and the quality of voice-hearer relationship: for instance, in case of feelings of inferiority and defectiveness concerning the self, this is mirrored in the voice being experienced as powerful, dominant, harming, and intrusive [52]. Given that induction of shame led to increased levels of intrusive thoughts and working memory impairments [34], there are reasons to expect that shame in response to a stressful event can be involved in the elicitation of AVH [53]. Therefore, shame can be not only simply correlated with this phenomenon, but it may be a causal factor in the onset of AVH [36]. One possible function of this process is proposed by McCarthy-Jones that evidenced that shame could elicit AVH to encourage submissive and self-protective behaviours avoiding situations that can be potentially threatening for one’s identity. For example, this can be true in people who lived negative past experiences of stigmatization, bullying or neglect. Previous studies have documented the occurrence of AVH following traumatic experiences in both Post Traumatic Stress Disorder (PTSD) [54–57] and dissociative disorders [58–60]. In this regard, the authors also found that the content of the voices was often symbolically or thematically related to the trauma but that the voices were distinguishable from memory representations of experiences, such as flashbacks [61]. Despite the intrinsic negative content related to shame, it’s possible that in healthy voice-hearers the way the message is communicated is different than in clinical voice hearers. The message might be reiterated in a less persistent way, or the same content might be expressed in a less directive way. For example, considering a given future social event, instead of voices that put the person in front of impending failures or experiences of inadequacy that will certainly happen, they might take the form of more friendly suggestions that give advice to avoid possible negative scenarios. Within this perspective, shame can be related to AVH in healthy voice-hearers playing an adaptive function that aids survival protecting people from further unpleasant experiences [36].

Finally, shame can in some occasions emerge because of AVH. The experience of AVH *per se* can constitute a significant source of stress for cultural reasons [62, 63]. Even when AVH is appraised by their hearing voice’s experience as benign, positive, and controllable, this phenomenon may be considered a social taboo, as it is difficult to talk about voices “without being looked at in a strange way” [24, 64, 65]. People who hear voices may report a lack of understanding by friends, families and even professionals and, as a result, many people hide these experiences, because of the fear of social judgment and stigma [65, 66]. People who experience hearing voices may feel themselves negatively judged by others and they can experience shame perceiving themselves as defective or inadequate [28]. Even though these experiences of isolation can still happen, it’s important to note that the stigma related to voice-hearing can be shaped by cultural and social conditions [67]. Voice-hearing embedded in an agreeable social world may become more manageable [68]. For those people who are part of social networks that provide support and validation the risk of being stigmatized is more contained. Furtherly, distressing voice-hearing may improve in response to specific coaching [69] and voice-hearing in religious contexts may become more culturally appropriate over time [70].

### Guilt and AVH in a non-clinical population

As with shame, guilt may influence people’s AVH. This can be common in people who experienced stressful life events that are linked with repentance or regret [36]. AVH with guilty content can be shown not only by veterans or people who experienced war or conflicts but also by people who experienced emotionally overwhelming events related to mistakes or bad actions. This voice represents the introjected negative thoughts and it ranges from unconscious or subliminal to fully conscious [71]. Guilty voices can be associated with emotional parts associated with self-criticizing narratives that can range from a benign advisor to a maladaptive judge [72]. In their worst expression, feelings of guilt can be a key driver to AVH related to past traumatic events (e.g. abuse, experiences of war or bullying) or be the content of intrusive and very disruptive AVH in schizophrenia [36]. Although their relationship with pathological disorders, guilty AVH can also have the role of establishing a precise responsibility related to personal negative experiences in (healthy) general population. Differently from clinical voice hearers, it is possible that the degree of inflexibility of guilty voices in healthy voice-hearers may be less intransigent and more ready to embrace a restorative perspective typical of an healthy guilt that can be resolved through reparation of the transgression. Therefore, AVH that show guilty contents in healthy voice hearers may also give a perception of order towards stressful events that otherwise would seem even more frightening and chaotic. This can give the transgressor a sense of control over negative events and help avoid similar mistakes in the future. AVH of guilt can be seen as an internal reminder of past mistakes that can be viewed as the source of helpful albeit challenging messages. Within this perspective, AVH of guilt can be related to an internalized voice of moral authority that can guide interpersonal behaviour enhancing social relationships and restoring equities [8].

### Shame and guilt AVH neuropsychological aspects

It is also relevant to point out that the relationships between shame, guilt and AVHs are linked to brain anatomy. Specifically, cognitive, and inhibitory functions, context binding, reality monitoring and metacognition play a relevant role in the development of AVHs. Their dysfunction may lead to the emergence of redundant and/or irrelevant or distractive information from the long-term memory to awareness, resulting in AVHs [39]. The negative emotional valence of AVH could be related to the activation of the right Broca’s area [46, 73]. In particular, AVHs were found to be similar to the automatic speech utterances that are generally generated by the right hemisphere of patients with aphasia. Owing to the role of Broca's area in the inhibitory process, its relationship with simple repetition is considered plausible [74]. Other studies noted a consistent activation of both amygdala and parahippocampal gyrus during processing the negative content of the heard voices [75]. These data are particularly consistent with other studies that found specific activations in the anterior cingulate cortex, parahippocampal gyrus related to shame and in the amygdala and insula for guilt [31].

Moreover, Van Lutterveld and colleagues found that, in resting-state networks, the role of the amygdala tends to decrease, and Vercammen found its reduced connectivity with the left temporoparietal region, which is recognised to be generally overactive during AVHs [76, 77].

De Boer and colleagues noted that individuals with AVHs tend to be less sensitive than the others in the auditory-verbal recognition and they show a higher false alarm rate if compared with people without AVHs. This may be related to an overload of the working memory that can be found within guilt and shame activations [34].

### Shame, guilt and AVH in psychotherapy

Even if the notion of hearing voices is not necessarily a pathological phenomenon, psychotherapy literature from very different orientations explored the multi-voiced nature of the self [78, 79]. Working on the inner voice of guilt and shame can be a very enriching experience within the therapy session, even when AVH don’t cause a pathological impairment. Different voices can be related to a different part of the self, some of which are more dominant than others, that may have different roles and relationships with each other’s [80]. AVH with guilty or shameful content can be related to a self-criticizing part of the self that even in a non-clinical population when is not properly accepted, can be extremely stressful. Within this context, psychotherapy can be the safe space where the therapist encourages clients to listen to their self-criticizing part and try to understand the reason for their shameful and guilty messages. This can start a new constructive dialogue between self-criticizing parts and other parts more related to compassion and non-judgment [81]. Clients can be encouraged to explore the content of that voices that are often tied to previous negative experiences that they can learn to manage and resolve [82]. Within this perspective, the therapist and the client start to give equal space to all the parts of the self-fostering the development of a richer and more integrated constellation of voices that express different parts of the client's identity [83].

### Limitations and strengths

The strength of this systematic review is its novelty. Indeed, no systematic review, to date, has had a focus on guilt and shame as related to auditory hallucinations in non-clinically impaired people. Nevertheless, the consistency of the results obtained, considering the variety of the type of articles included, the research design, the instruments adopted, and the statistical analyses carried out, prevents generalizability. Moreover, the sample size of these studies is small and often includes great heterogeneity in terms of age and gender, as well as no follow-up.

The present review focused only on shame and guilt and does not aim to include all the nuances and the diversity of positive voice experiences that can occur in the general population. Future research is needed to explore the relationship between AVHs and other emotional experiences.

Other limitations of this review concern the search strategy. Although our literature search was conducted in eight databases and several search terms were applied, the search may not be exhaustive. Eligible studies are only from 7 countries and most of them were carried out in the USA.

It’s also important to highlight that the present systematic review didn’t consider the relationship between AVHs, guilt and shame in the clinical population. The consideration of this kind of population could be the subject of subsequent studies. In this regard, as evidenced by recent findings [84], it may be also interesting to look at socio-economic variables or the presence of risky behaviors. The role of drug use, such as cannabis, in the onset of AVHs, in similar psychosocial conditions, in people who do not need treatment and those who do could be key factors to be considered in future reviews.

### Implications for practice and research

Most studies included in our review focused on shame or guilt and not on both, and this makes it complex to examine whether they do not also influence the occurrence of AVHs together or arise together. Psychological interventions are suggested to target maladaptive appraisals and allow the construction of affect regulation strategies, including shame and guilt, which may play an important role in increasing the distress associated with AVHs, in those who are not clinically impaired. In addition, longitudinal studies with long follow-ups would be essential to highlight the direction of the relationship between AVHs, shame and guilt. Thus, there is a need to establish the longer-term association of shame, guilt, and hearing voices. As well their effects on long term mental health outcomes and hearing voices themselves, shame and guilt may contribute to adverse consequences such as isolation or increasing rates of depression, anxiety, or other mental disorders.

Our systematic review shows that there are patterns whereby emotions such as guilt and shame are used as a resource by non-clinical voice hearers. The therapist could work in the non-clinical population so that, in order to prevent and/or avert negative consequences deriving from the overriding experience of emotions such as guilt and shame, often more derived from the label of 'psychosis' or 'psychotic symptom', these same emotions can be used to build a protective network that averts the need for more intense treatment [41, 85–87]. It is possible for the person hearing a voice of guilt or shame to reprocess the heard message adaptively and functionally on their own. Therefore, voice-hearing of shame and guilt, as of other emotions, is part of human experience, which only in some cases, may be associated with distress and need for care. If the discomfort associated with hearing voices in those considered to belong to the 'non-clinical' population is high enough to require treatment (e.g., psychotherapy), we should consider this to be a 'risk' population. A possible therapeutic intervention related to the common experience of voice hearing could be to help the client embrace this voice, carefully consider the shameful or guilty proposed issues, and work to promote in the client a flexible and constructive discussion with the voice itself. In this regard, these aspects could be further explored in future studies, outlining treatments that allow for the restructuring of the use of these same emotions as a buffer against, for example, experienced stigma and/or potential negative development. Indeed, more systematic exploration is needed about how shame and guilt affect hearing voices in non-clinical people and vice versa. Future research could focus on both clinical and non-clinical voice-hearers that often deal with shameful or guilty messages. Frequency in voice-hearing, the presence of life-stressors, as well as the non-directive vs hostile nature of the voices may be considered as key factors to gather evidence helping support the solidity of a quasi- vs a fully-dimensional model.

## Conclusions

In producing this report, we were constrained by the relative scarcity of well-powered studies to conduct a systematic review of the literature, which is why we elected to produce a narrative review. This review is intended to provide an overview of the relationships between AVHs, shame and guilt in a non-clinical sample. Even if these two self-conscious emotions can be easily found as a common topic in a lot of AVHs, other unpleasant emotional experiences should also be explored in future research.s

Future studies should endeavour to recruit larger cohorts to further examine the potential and reciprocal influences between AVHs, shame and guilt as well as to identify benefits and adverse events of possible therapies.

## Supplementary Information

**Additional file 1: Supplementary Material 1.** Criteria for quality assessment and study evaluation table

## Data Availability

All data generated or analysed during this study are included in this published article [and its supplementary information files].

## Abbreviations

AVHs: Auditory Verbal Hallucinations
PTSD: Post Traumatic Stress Disorder
MMAT: Mixed Methods Appraisal Tool
